# Supplementary material for: A signal cascade originated from epidermis defines apical-basal patterning of Arabidopsis shoot apical meristems
Source: Nat Commun. 2020 Mar 5;11:1214. doi: 10.1038/s41467-020-14989-4 (PMC7058014; doi:10.1038/s41467-020-14989-4)
Supplement: Supplementary file 1 — Supplementary Information [file 41467_2020_14989_MOESM1_ESM.pdf]

Supplementary Information For

A signal cascade originated from epidermis defines apical-basal patterning of Arabidopsis shoot apical meristems

Han et al.,

Supplementary Figures 1 - 18

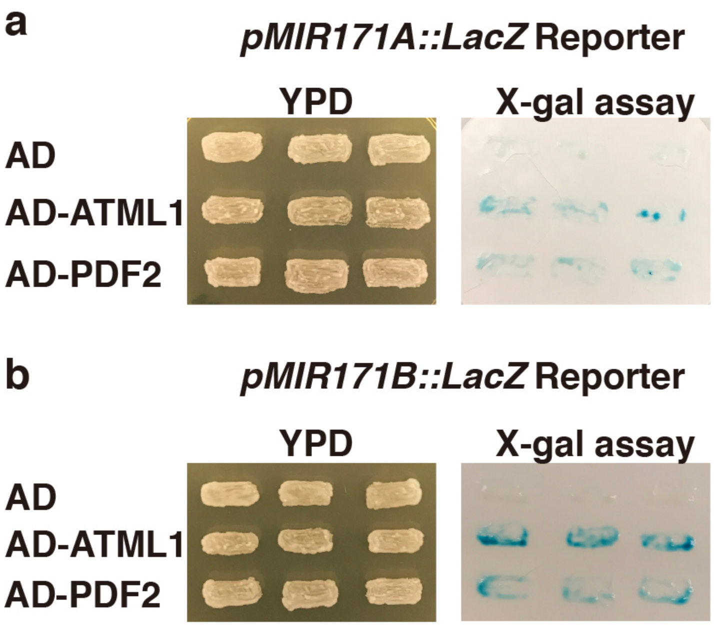

Supplementary Figure 1. Y1H X-gal assays show that ATML1 or PDF2 interacts with the promoters of both *MIR171A* (a) and *MIR171B* (b). The left panel: three independent yeast transformants grown on the YPD plates; the right panel: x-gal lifting assays using the yeast cells shown on the left panel. Blue color indicates the interaction between proteins and the promoter DNAs. The left panel and right panel are mirror symmetric.

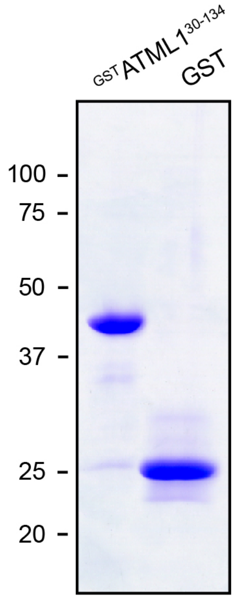

Supplementary Figure 2. The ^{GST}ATML1$^{30\text{-}134}$ and GST protein used for EMSA. Purified recombinant ^{GST}ATML1$^{30\text{-}134}$ protein or GST protein were fractionated on an SDS-PAGE gel and visualized with Coomassie blue staining. Source data are provided as a Source Data file.

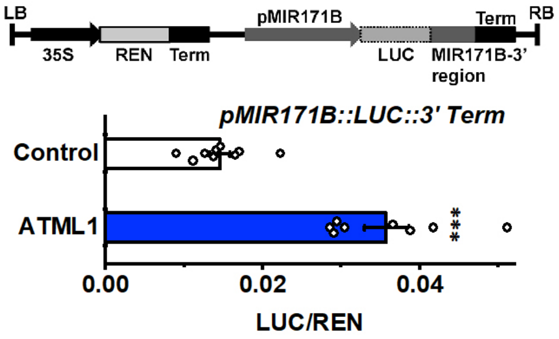

Supplementary Figure 3. The dual-luciferase assay shows that ATML1 activates the expression of *pMIR171B::LUC::3’ terminator*, a *MIR171B* luciferase reporter. Upper panel: the structure of the reporter used in the dual-luciferase assay. The 3’ terminator of *MIR171B* used here is identical to the one used for constructing the *pMIR171B::H2B-GFP* reporter in Figure 1. Lower panel: ratio of firefly luciferase (LUC) to Renilla luciferase (REN) activity in tobacco cells co-transformed with the *pMIR171B::LUC:: 3’ terminator* reporter and the ATML1 effector (n=9 biological replicates) is significantly higher than that in cells co-transformed with the same reporter and empty vector control (n=8 biological replicates). Bar: mean $\pm$ SE. ***P<0.001 (Student’s two-tailed t-test). Source data are provided as a Source Data file.

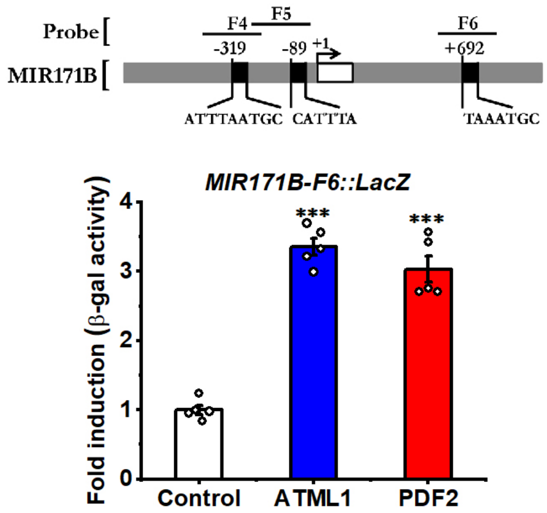

Supplementary Figure 4. Y1H assays show that ATML1 and PDF2 proteins interact with the fragment 6 (F6) from the 3' terminator of the *MIR171B* gene. The upper panel: schematic structure of the *MIR171B* gene. The expanded diagram shows the sequences of putative L1 boxes on the gene. Probes indicate the DNA fragments used in the Y1H assay. +1 indicates the transcription start site. The lower panel: the relative activity of the *pMIR171B-F6::lacZ* reporter. All the numbers are normalized to the average value of the empty vector control. Bar: mean ± standard error (SE) (n=5 biological replicates). ***P<0.001 (Student's two-tailed t-test). Source data are provided as a Source Data file.

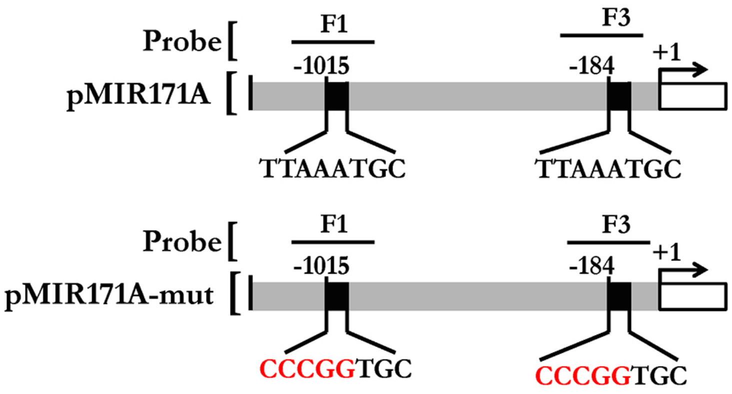

Supplementary Figure 5. Schematic structure of the *pMIR171A* and the *pMIR171A-mut*, which are used in the dual-luciferase assays, Y1H and the transgenic studies in Figure 5. Upper panel: The expanded diagram showed the sequences of putative L1 boxes in the promoters. Probes indicate the DNA fragments used in the EMSA and the Y1H assay. Lower panel: schematic structure of the promoter of *MIR171A* with two mutated L1 binding sites (named as *pMIR171A-mut*) used in the dual-luciferase assays, Y1H and the transgenic studies. Mutated nucleotides in the L1 boxes are indicated in red. +1 indicates the transcription start site.

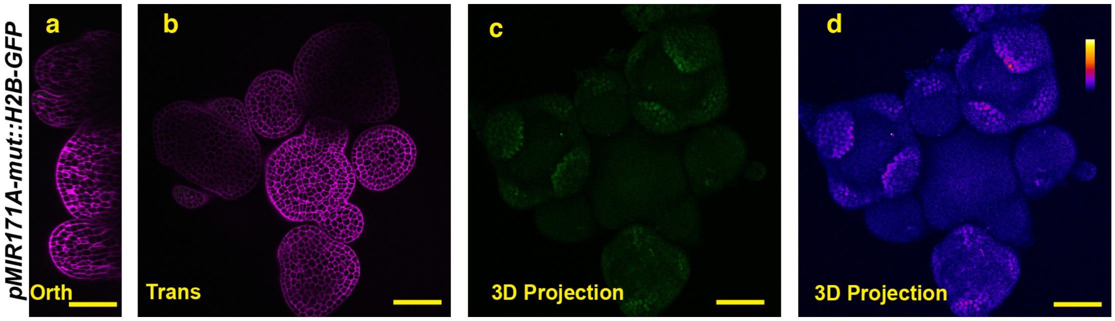

Supplementary Figure 6. (a-d) Expression of *pMIR171A-mut::H2B-GFP* in the SAM from a different independent transgenic line (line 15). The *Arabidopsis* SAM is shown from orthogonal view (a), in the transverse optical section in corpus (b), and from 3D projection view (c, d). The confocal settings for imaging GFP in (a-d) are identical to that in Fig. 5 d-k, and the quantification of GFP is indicated by the identical color bar in (Fig. 5 g, k). Panels: (a-b): merge of GFP (green) and PI counterstain (purple); (c) GFP (green); (d): GFP quantified from c (quantification indicated by color). Scale bar: 20 µm; color bar (d): fire quantification of signal intensity.

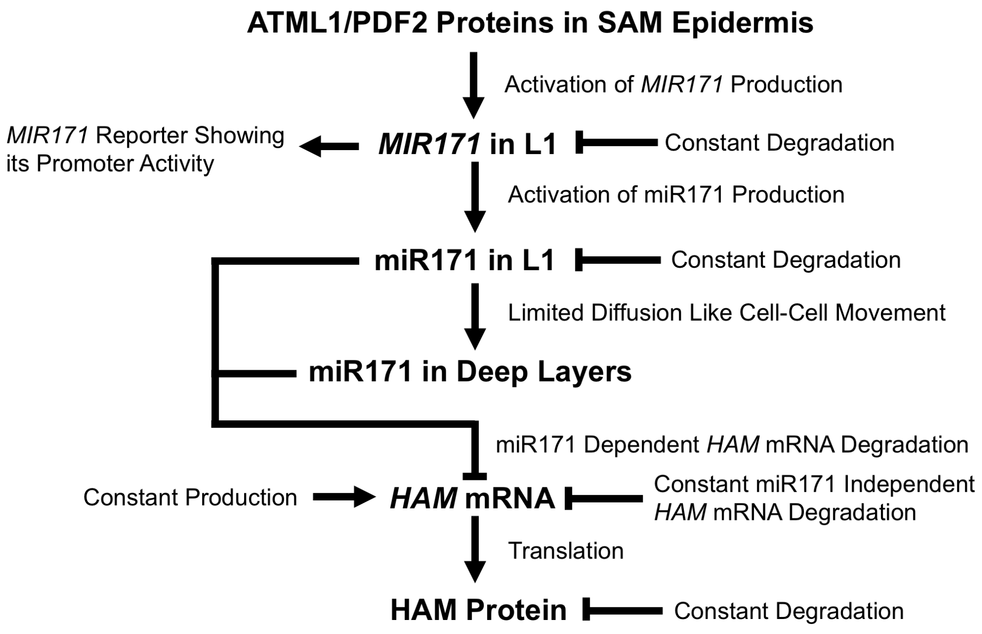

Supplementary Figure 7. Schematic representation of the computational model simulating the L1-miR171-HAM signaling cascade in the SAM. Arrows represent positive terms and blunt arrows represent negative terms. Details of the computational model are described in the methods.

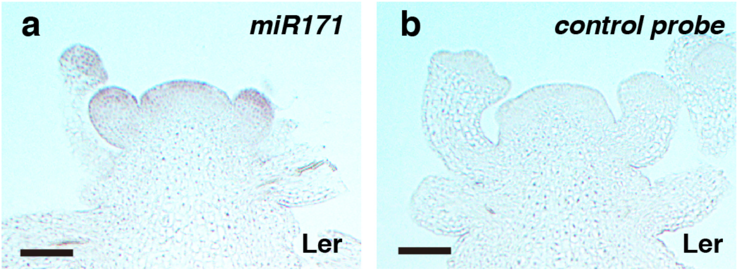

Supplementary Figure 8. Localization of miR171 RNAs in the inflorescence SAMs from L*er* wild type plants. (a) RNA *in situ* hybridization was performed in the inflorescence SAM using the miR171 miRCURY LNA Detection probe, which specifically recognizes Arabidopsis miR171 (See methods for details). (b) RNA *in situ* hybridization as performed in the inflorescence SAM using miR124 miRCURY LNA detection probe, which specifically recognizes murine miR124, serving as a negative control (See methods for details). The experiment for (a) and (b) was performed at the same time using the identical procedure. Four independent biological replicates for each probe were performed with similar results. Scale bar: 50 µm.

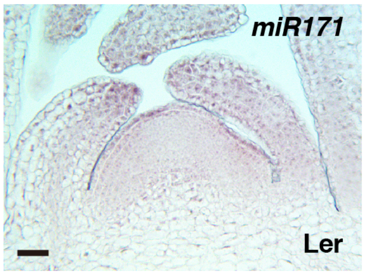

Supplementary Figure 9. Localization of miR171 RNAs in the vegetative SAM from the L*er* wild type. RNA *in situ* hybridization was performed in the vegetative SAM using the miR171 miRCURY LNA Detection probe (See methods for details). Five independent biological replicates were performed with similar results. Scale bar: 10 μm.

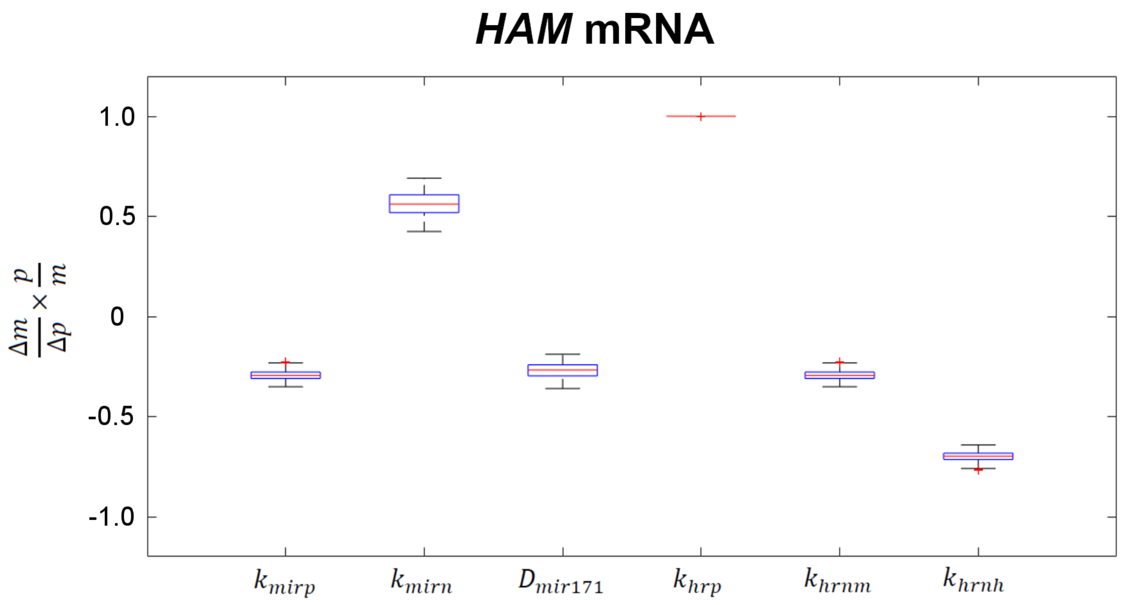

Supplementary Figure 10. Sensitivity analysis of the six key parameters in the computational model, using 235 sets of parameter values. For *HAM* mRNA, the sensitivity of the 235 sets of the parameter values (listed in Supplementary Data 1) for the six key parameters is plotted with boxplots. On each box, the central line: the median; the bottom and top edges of the box: the 25th and 75th percentiles, respectively. The whiskers: the most extreme data points not considered outliers; the + symbol: the individually plotted outlier. Source data are provided as a Source Data file.

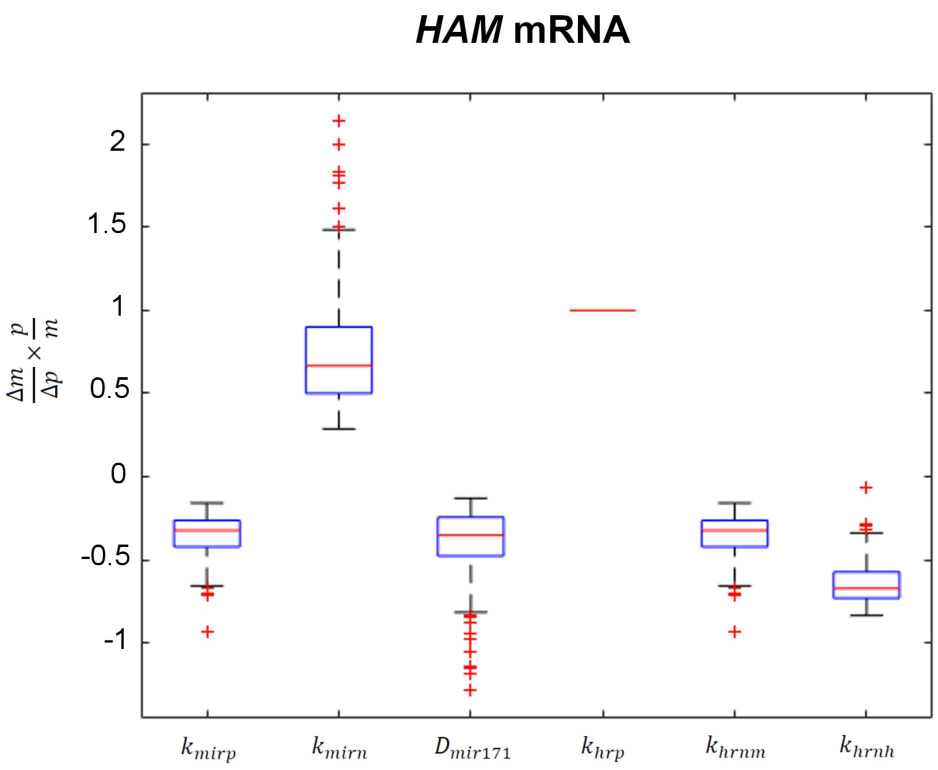

Supplementary Figure 11. Sensitivity analysis of the six key parameters in the computational model, using 173 sets of randomly selected parameter values. For *HAM* mRNA, the sensitivity of the 173 sets of randomly selected parameter values (listed in Supplementary Data 3) for the six key parameters is plotted with boxplots. On each box, the central line: the median; the bottom and top edges of the box: the 25th and 75th percentiles, respectively. The whiskers: the most extreme data points not considered outliers; the + symbol: the individually plotted outlier. Source data are provided as a Source Data file.

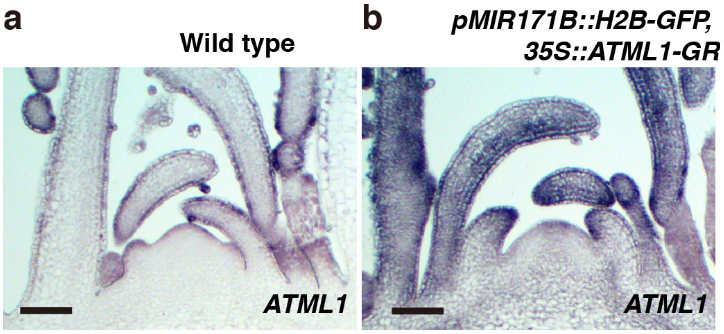

Supplementary Figure 12. RNA *in situ* hybridization of *ATML1* mRNA in wild type (a) and the *35S::ATML1-GR*; *pMIR171B::H2B-GFP* plant (b). Compared to the wild type control (a), *ATML1* is overexpressed but not uniformly expressed in cells of the SAM from the *35S::ATML1-GR*; *pMIR171B::H2B-GFP* plant (b). The experiment was performed using the identical procedure. Three biological replicates were performed with similar results. Scale bar: 50 µm.

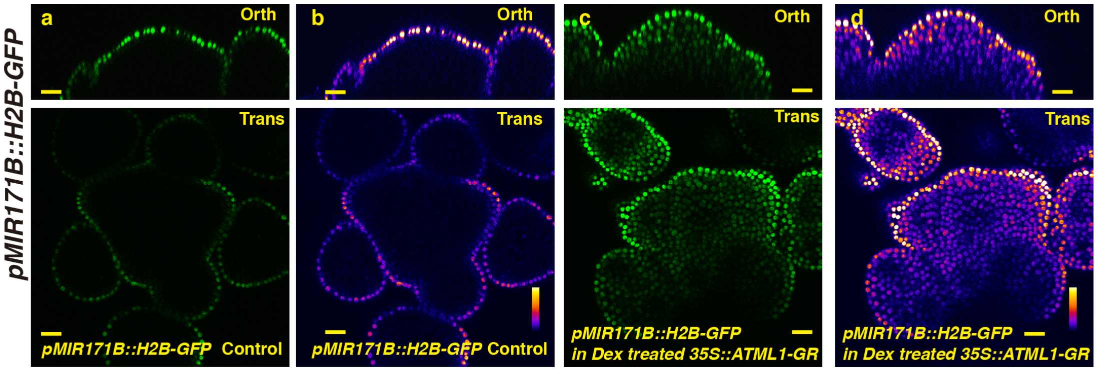

Supplementary Figure 13. *MIR171B* is induced by ATML1 in Arabidopsis. (a-d) Confocal live imaging of the *pMIR171B::H2B-GFP* reporter in the SAMs without the Dex treatment (a,b) or at 9 days after the Dex treatment of the *35S::ATML1-GR* plants (c,d). (a, c): GFP (green); (b, d): quantified GFP (quantification indicated by color); color bar: fire quantification of signal intensity. Top panel: orthogonal view; bottom panel: transverse optical section view in corpus. Scale bar: 20 μm.

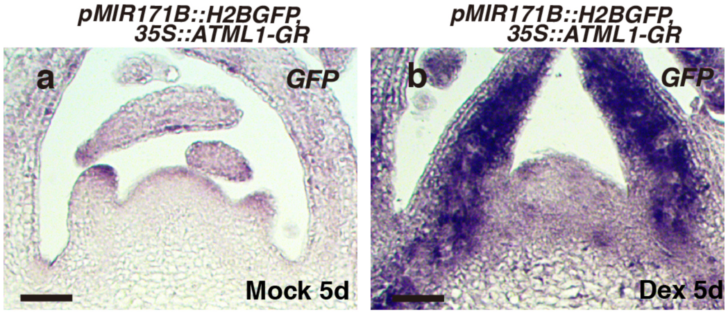

Supplementary Figure 14. RNA *in situ* hybridization of *GFP* mRNA showed the activation of *MIR171B* after the Dex treatment in the SAMs and leaves of the *35S::ATML1-GR*; *pMIR171B::H2B-GFP* plant. (a) A mock treatment as the control and (b) five days after the Dex treatment. The experiment was performed using the identical procedure. Two biological replicates were performed with similar results. Scale bar: 50 µm.

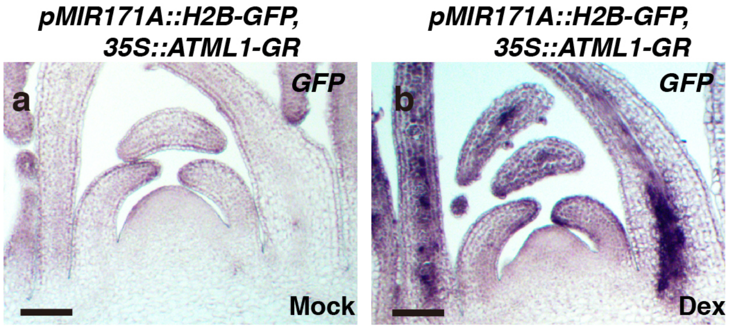

Supplementary Figure 15. RNA *in situ* hybridization of *GFP* mRNA showed the activation of *MIR171A* after the Dex treatment in the SAMs and leaves of the *35S::ATML1-GR*; *pMIR171A::H2B-GFP* plant. (a) A mock treatment as the control and (b) 24 hours after the Dex treatment. The experiment was performed using the identical procedure. Three biological replicates were performed with similar results. Scale bar: 50 µm

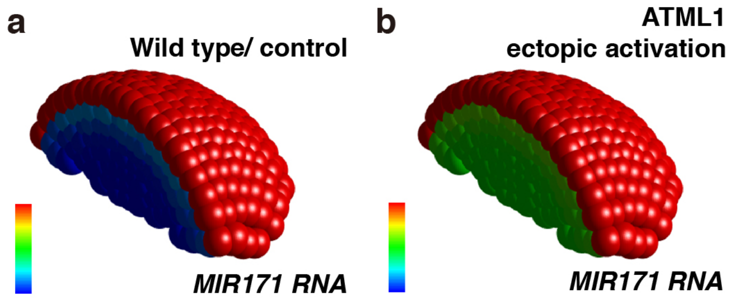

Supplementary Figure 16. Simulated miR171 RNA levels in the SAMs of the wild type control plants (a) and the *ATML1* ectopic activation plants (b). In each cell, the simulated miR171 RNA level is indicated by color, with the gradient from blue (0) to red (at or above 0.8 a.u.).

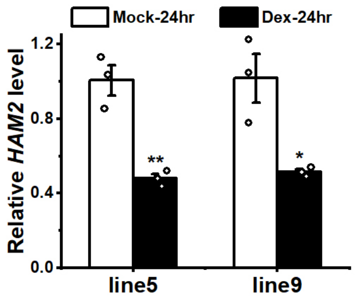

Supplementary Figure 17. Quantification of the *HAM2* transcript levels in two independent *35S::ATML1-GR; pMIR171B::H2B-GFP* transgenic lines (#5 and #9) 24 hours after mock or the Dex treatment. Bars: mean ± SE (*n*=3 biological replicates). **P<0.01,*P<0.05 (Student's two-tailed t-test). Source data are provided as a Source Data file.

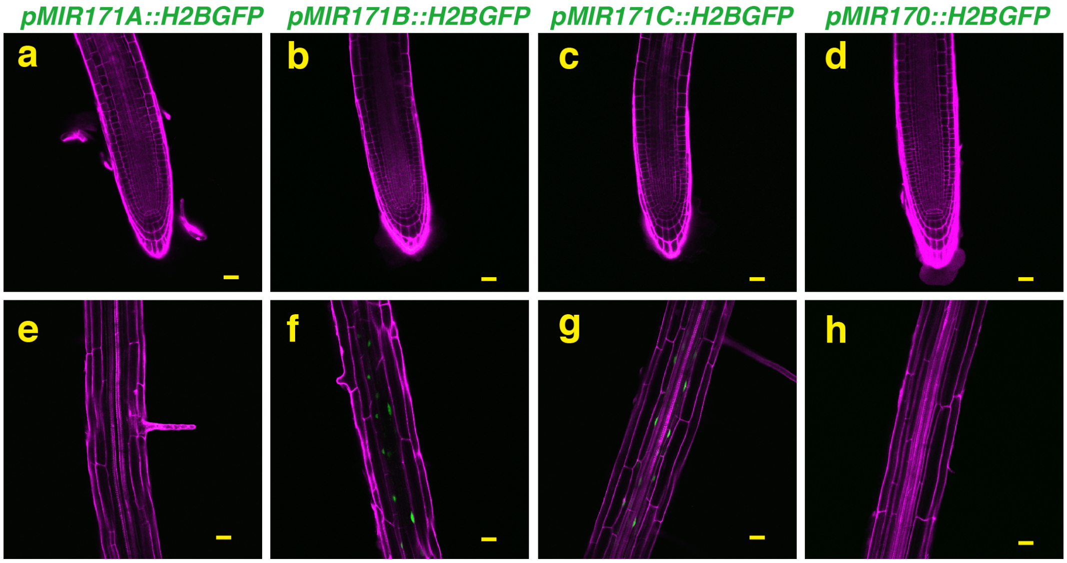

Supplementary Figure 18. The expression patterns of different *MIR171/170* genes in the root. (a-d) The *pMIR171A::H2B-GFP* (a), *pMIR171B::H2B-GFP* (b), *pMIR171C::H2B-GFP* (c) or *pMIR170::H2B-GFP* (d) is not expressed in the root apical meristem. (e-h) *pMIR171A::H2B-GFP* (e) and *pMIR170::H2B-GFP* (h) is not expressed in the differentiation zone of the root. *pMIR171B::H2B-GFP* (f) and *pMIR171C::H2B-GFP* (g) is expressed in the differentiation zone of the root. Seedlings were grown on half MS plates in the short day condition for 10 days. The expression patterns of these reporters were confirmed with two independent lines and with at least three biological replicates for each line. Green: H2B-GFP, Purple: PI. Scale bar: 25 μM.

Supplementary Tables 1-2

**Parameter**	**Biological Meaning**	**Value**	**Unit**
k_{mirp}	Parameter for miR171 production	1	/h
k_{mirn}	Parameter for miR171 degradation	1	/h
D_{mir171}	Rescaled diffusion constant for miR171 movement	0.012	area a.u./h
k_{hrp}	Parameter for constant *HAM* mRNA production	0.6	concentration a.u./h
k_{hrnm}	Parameter for miR171 dependent *HAM* mRNA degradation	6	/(concentration a.u.·h)
k_{hrnh}	Parameter for miR171 independent *HAM* mRNA degradation	0.24	/h
k_{hpp}	Parameter for HAM protein production	0.5	/h
k_{hpn}	Parameter for HAM protein degradation	0.5	/h
k_{grp}	Parameter for *pMIR171::H2B-GFP* reporter mRNA production	1	/h
k_{grn}	Parameter for *pMIR171::H2B-GFP* reporter mRNA degradation	1	/h
k_{gpp}	Parameter for *pMIR171::H2B-GFP* reporter protein production	1	/h
k_{gpn}	Parameter for *pMIR171::H2B-GFP* reporter protein degradation	1	/h

Supplementary Table 1. One set of parameters for the SAM model.

Parameter	Parameter Ranges	Unit
k_{mirp}	0.5-1.5	/h
k_{mirn}	0.8-1.4	/h
D_{mir171}	0.006-0.018	area a.u./h
k_{hrp}	0.528-0.78	concentration a.u./h
k_{hrnm}	3.36-9.24	/(concentration a.u.·h)
k_{hrnh}	0.12-0.2832	/h

Supplementary Table 2. Ranges of parameter values for one set of six key parameters (shown in Supplementary Table 1) for different solutions.
