# Supplementary material for: A signal cascade originated from epidermis defines apical-basal patterning of Arabidopsis shoot apical meristems
Source: Nat Commun. 2020 Mar 5;11:1214. doi: 10.1038/s41467-020-14989-4 (PMC7058014; doi:10.1038/s41467-020-14989-4)
Supplement: Supplementary file 3 — Description of Additional Supplementary Files [file 41467_2020_14989_MOESM3_ESM.pdf]

**Description of Additional Supplementary Files**

File Name: Supplementary Data 1
Description: Parameter values (235 sets) of the six key parameters. These values lead to simulated HAM gene expression patterns qualitatively comparable to experimental observations.

File Name: Supplementary Data 2
Description: Random parameter values (20,000 sets) for six key model parameters for the screening of possible solutions.

File Name: Supplementary Data 3
Description: Random parameter values (173 sets from the 20,000 sets in Supplementary Data 2) for the six key model parameters. These values lead to simulated HAM gene expression patterns qualitatively comparable to experimental observations.

File Name: Supplementary Movie 1
Description: Full stacks of confocal image sections of the pMIR171A::H2B-GFP reporter (green) in the same wild type SAM as in Fig. 1, with cellular outlines (purple) stained with propidium iodide (PI), from the bottom to the top.

File Name: Supplementary Movie 2
Description: Full stacks of confocal image sections of the pMIR171B::H2B-GFP reporter (green) in the same wild type SAM as in Fig. 1, with cellular outlines (purple) stained with propidium iodide (PI), from the bottom to the top.

File Name: Supplementary Movie 3
Description: Full stacks of confocal image sections of the pMIR171C::H2B-GFP reporter (green) in the same wild type SAM as in Fig. 1, with cellular outlines (purple) stained with propidium iodide (PI), from the bottom to the top.

File Name: Supplementary Movie 4
Description: Full stacks of confocal image sections of pMIR170::H2B-GFP reporter (green) in the same wild type SAM as in Fig. 1, with cellular outlines (purple) stained with propidium iodide (PI), from the bottom to the top.

File Name: Supplementary Movie 5
Description: Full stacks of confocal image sections of pMIR170::H2B-GFP reporter (green) in the same wild type SAM as in Fig. 1, with cellular outlines (purple) stained with propidium iodide (PI), from the bottom to the top.

File Name: Supplementary Movie 6
Description: Full stacks of confocal image sections of pHAM2::H2B-GFP reporter (green) in the same wild type SAM as in Fig. 1, with cellular outlines (purple) stained with propidium iodide (PI), from the bottom to the top.

File Name: Supplementary Movie 7
Description: Simulated steady stage solutions of HAM mRNA levels in SAMs when kmirp is varied (values indicated in movie). Standard values listed in Supplementary Table 1 are assigned to model

parameters other than kmirp. In each cell, the simulated HAM mRNA level is indicated by color, with the gradient from blue (0) to red (at or above 2 a.u.).

File Name: Supplementary Movie 8
Description: Simulated steady stage solutions of HAM mRNA levels in SAMs when kmirn is varied (values indicated in movie). Standard values listed in Supplementary Table 1 are assigned to model parameters other than kmirn. In each cell, the simulated HAM mRNA level is indicated by color, with the gradient from blue (0) to red (at or above 2 a.u.).

File Name: Supplementary Movie 9
Description: Simulated steady stage solutions of HAM mRNA levels in SAMs when Dmir171 is varied (values indicated in movie). Standard values listed in Supplementary Table 1 are assigned to model parameters other than Dmir171. In each cell, the simulated HAM mRNA level is indicated by color, with the gradient from blue (0) to red (at or above 2 a.u.).

File Name: Supplementary Movie 10
Description: Simulated steady stage solutions of HAM mRNA levels in SAMs when khrp is varied (values indicated in movie). Standard values listed in Supplementary Table 1 are assigned to model parameters other than khrp. In each cell, the simulated HAM mRNA level is indicated by color, with the gradient from blue (0) to red (at or above 2 a.u.).

File Name: Supplementary Movie 11
Description: Simulated steady stage solutions of HAM mRNA levels in SAMs when khrnm is varied (values indicated in movie). Standard values listed in Supplementary Table 1 are assigned to model parameters other than khrnm. In each cell, the simulated HAM mRNA level is indicated by color, with the gradient from blue (0) to red (at or above 2 a.u.).

File Name: Supplementary Movie 12
Description: Simulated steady stage solutions of HAM mRNA levels in SAMs when khrnh is varied (values indicated in movie) Standard values listed in Supplementary Table 1 were assigned to model parameters other than khrnh. In each cell, the simulated HAM mRNA level is indicated by color, with the gradient from blue (0) to red (at or above 2 a.u.).

File Name: Supplementary Movie 13
Description: Simulated steady stage solutions of HAM mRNA levels in SAMs using 173 sets of parameters randomly searched from 20,000 sets of random parameters (Supplementary Data 2, Supplementary Data 3). Parameter values for each set of solutions are listed in Supplementary Data 3. In each cell, the simulated HAM mRNA level is indicated by color, with the gradient from blue (0) to red (at or above 2 a.u.).

File Name: Supplementary Movie 14
Description: Simulated steady stage of the HAM mRNA levels in SAMs when ATML1 is ectopically activated, using 26 sets of different ML1p inputs. ML1p (functional ATML1 and PDF2 proteins) inputs in different layers of the SAM are listed in the movie. In each cell, the simulated HAM mRNA level is indicated by color, with the gradient from blue (0) to red (at or above 2 a.u.).

File Name: Supplementary Movie 15

Description: Simulated steady stage of the HAM mRNA levels in SAMs whenATML1 is ectopically activated, using 50 additional sets of different ML1p inputs. ML1p (functional ATML1 and PDF2 proteins) inputs in different layers of the SAM are listed in the movie. In each cell, the simulated HAM mRNA level is indicated by color, with the gradient from blue (0) to red (at or above 2 a.u.).

File Name: Supplementary Movie 16
Description: Full stacks of confocal image sections of the pMIR171B::H2B-GFP reporter (green) through the differentiation zone of the same wild type root shown in Fig. S18 f, with cellular outlines (purple) stained with propidium iodide (PI).

File Name: Supplementary Movie 17
Description: Full stacks of confocal image sections of the pMIR171C::H2B-GFP reporter (green) through the differentiation zone of the same wild type root shown in Fig. S18 g, with cellular outlines (purple) stained with propidium iodide (PI).
